# Medical College Notes

**Published:** 1894-11

**Authors:** 


### College Notes.

THE Cincinnati College of Medicine and Surgery (medical department of the University of Cincinnati,) has issued the announcement for its forty-fourth year. There are some features of this pamphlet that deserve special comment. In the first place, we find on page 20 a summary of the existing state laws relating to the practice of medicine, which closes with this significant sentence, "other states will probably soon enact similar laws."

Under the head of requirements for admission is published a blank form to be filled out by the candidate; and a note is added requiring the statements therein as to qualification to be confirmed by appropriate vouchers.

A further distinguishing feature of this announcement is, that eminent members of the medical profession throughout the country will address the students on special topics. The list embraces: Dr. Donald Maclean, of Detroit; Dr. Joseph Price, of Philadelphia; Dr. John R. Van Hoff, of U. S. Army; Dr. L. S. McMurtry, of Louisville; Dr. Isaac N. Love, of St. Louis; Dr. Lawrence C. Carr, of Cincinnati; Dr. Karl von Ruck, of Asheville.

Finally, a number of prizes are offered which tempt a friendly rivalry in the several departments of medicine.

The elevated standard which this school has adopted is an index of the improved environment of medical college teaching all over the land.

---

THE faculty of the medical department of the University of Buffalo announces that a course of ten lectures on the History of Medicine will be given in Alumni Hall at the college building by Dr. Roswell Park. The first lecture was delivered Monday evening, October 8, 1894, and subsequent lectures will occupy Monday evenings until the course is completed. These lectures are open to all who are interested in the subject.

---

TAYLOR BROTHERS COMPANY, of Rochester, N. Y., have issued a neat little circular giving information on the subject of clinical thermometers, which will be sent on application. Every doctor knows the importance of accuracy in clinical thermometers, and no physician has yet complained that Taylor's certified thermometers are not of the best.

